# 

**Published:** 1910-12

**Authors:** 


### The Library of the
### Bristol Medico-Chirurgical Society.

---

*The following donations have been received since the publication of the List in September.*

*November 30th,* 1910.

Fleet-Surgeon W. E. Home (1) .. .. 1 volume.
L. M. Griffiths .. .. .. .. 2 framed pictures.

### SEVENTY-EIGHTH LIST OF BOOKS.

The titles of books mentioned in previous lists are not repeated.

The figures in brackets refer to the figures after the names of the donors, and show by whom the volumes were presented. The books to which no such figures are attached have either been bought from the Library Fund, or received through the *Journal.*

| | | |
|---|---|---|
| **Aarons, S. J.** .. | *Gynæcological Therapeutics* .. .. .. .. | 1910 |
| **Allbutt and H. D. Rolleston, Sir C.** [Eds.]. | *A System of Medicine.* Vol. VIII | 1910 |
| **Arderne, John** .. | *Treatises of Fistula in Ano, Hæmorrhoids, and Clysters* (Ed. by D'A. Power) .. [Reprint] | 1910 |
| **Ashby, H.** .. .. | *Notes on Physiology* (Ed. by H. T. Ashby). 8th Ed. | 1910 |
| **Bell, W. B.** .. | *The Principles of Gynæcology* .. .. .. .. | 1910 |
| **Bidwell, L. A.** .. | *Intestinal Surgery* .. .. .. .. 2nd Ed. | 1910 |
| **Brown, W. L.** .. | *Physiological Principles in Treatment.* 2nd Ed. | 1910 |
| **Charpentier, A. E. L.** | *Hæmoglobinuria* .. .. .. .. .. .. .. | 1910 |
| **Daniels, C. W.** .. | *Tropical Medicine and Hygiene* .. Pt. II | 1910 |
| **Fein, J.** .. .. | *Hints in Rhinology and Laryngology* (Tran. by J. B. Horgan) .. .. .. .. .. .. .. | 1910 |
| **Fortescue-Brickdale, J. M.** | *A Practical Guide to the Newer Remedies* | 1910 |
| **Giles, A. E.** .. | *After-results of Abdominal Operations* .. .. | 1910 |
| **Haas, P.** .. .. | *Organic Chemistry* .. .. .. .. .. .. .. | 1910 |
| **Hart, D. B.**.. .. | *Phases of Evolution and Heredity* .. .. .. | 1910 |
| **Hollander, B.** .. | *The Mental Symptoms of Brain Disease* .. .. | 1910 |
| **Horder, T. J.** .. | *Clinical Pathology in Practice* .. .. .. .. | 1910 |
| **Kidd, F.** .. .. | *Urinary Surgery* .. .. .. .. .. .. .. .. | 1910 |
| **Lea, A. W. W.** .. | *Puerperal Infection* .. .. .. .. .. .. .. | 1910 |
| **Lejars, F.** .. .. | *Urgent Surgery* (Tran. by W. S. Dickie) Vol. II | 1910 |
| **Maddox, E. E.** .. | *Golden Rules of Refraction* .. .. .. 3rd Ed. | [N.D.] |
| **Marsh, H.** .. .. | *Diseases of the Joints and Spine* (Ed. by C. G. Watson) .. .. .. .. .. .. .. 3rd Ed. | 1910 |
| **Moon, R. O.** .. | *The Relation of Medicine to Philosophy* .. (1) | 1909 |
| **Morison, R.**.. .. | *An Introduction to Surgery* .. .. .. .. .. | 1910 |
| **Morre, H.** [Ed.] | *Medical Guide to Watering Places and Sanatoria* 2nd Ed. | 1910 |

| | | |
|---|---|---|
| **Murray, R. W.** .. | *Hernia* .. .. .. .. .. .. .. .. 2nd Ed. | 1910 |
| **Neuburger, M.** .. | *History of Medicine* (Tran. by E. Playfair) Vol. I | 1910 |
| **Peckham, F. E.** | *The Classification and Treatment of Diseases commonly known as Rheumatism* .. .. .. | 1910 |
| **Pringle, J. H.** .. | *Fractures and their Treatment* .. .. .. .. | 1910 |
| **Pye [W.]** .. .. | *Elementary Bandaging and Surgical Dressing* (Ed. by W. H. Clayton-Greene and V. Z. Cope) .. .. .. .. .. .. .. 12th Ed. | 1910 |
| **Rolleston, Sir C. Allbutt and H. D.** [Eds.]. | *A System of Medicine* Vol. VIII | 1910 |
| **Schafer, E. A.** .. | *Essentials of Histology* .. .. .. 8th Ed. | 1910 |
| **Sprigge, S. S.** .. | *Some Considerations of Medical Education* .. | 1910 |
| **Sutherland, W. G.** | *Dispensing made Easy* (Revised by F. J. Warwick).. .. .. .. .. .. 4th Ed. | 1910 |
| **Weiss, R.** .. .. | *Manual of Clinical Pathology* .. .. .. .. | 1910 |
| **Whitelocke, R. H. A.** | *Sprains and Allied Injuries to Joints* 2nd Ed. | 1910 |
| **Wootton, A. C.** .. | *Chronicles of Pharmacy* .. .. .. 2 Vols. | 1910 |
| **Yonge, E. S.** .. | *Hay Fever and Paroxysmal Sneezing* .. .. | 1910 |

### TRANSACTIONS, REPORTS, JOURNALS, &c.

| | | |
|---|---|---|
| American Association of Obstetricians and Gynecologists, Transactions of the .. .. .. .. .. .. .. .. .. .. | Vol. XXII | 1910 |
| American Journal of the Medical Sciences, The | Vol. CXXXIX | 1910 |
| American Laryngological Association, Transactions of the .. .. | | 1910 |
| Archives of the Middlesex Hospital .. .. .. .. | Vol. XIX | 1910 |
| Boston Medical and Surgical Journal .. .. .. .. | Vol. CLXII | 1910 |
| British Medical Journal, The .. .. .. .. .. .. .. | Vol. I for | 1910 |
| College of Physicians of Philadelphia, Transactions of the | Vol. XXXI | 1909 |
| Edinburgh Obstetrical Society, The Transactions of the | Vol. XXXV | 1910 |
| Edinburgh Medical Journal .. .. .. .. .. .. .. | N.S., Vol. IV | 1910 |
| Gazette des Hôpitaux .. .. .. .. .. .. .. .. .. .. .. .. | | 1909 |
| Glasgow Medical Journal, The .. .. .. .. .. | Vol. LXXIII | 1910 |
| Hospital, The .. .. .. .. .. .. .. .. .. .. .. | Vol. XLVII | 1910 |
| Journal of Medical Research, The .. .. .. .. .. | Vol. XXII | 1910 |
| Journal of Obstetrics and Gynæcology, The .. .. | Vol. XVII | 1910 |
| Journal of the American Medical Association, The .. | Vol. LIV | 1910 |
| Lancet, The .. .. .. .. .. .. .. .. .. .. .. | Vol. I for | 1910 |
| Münchener medicinische Wochenschrift .. .. .. | Bd. I für | 1910 |
| Practitioner, The .. .. .. .. .. .. .. .. .. | Vol. LXXXIV | 1910 |
| Progressive Medicine .. .. .. .. .. .. .. .. .. | Vol. III | 1910 |
| Revue hebdomadaire de Laryngologie .. .. | Tom. XXX, Vol. I | 1910 |
| St. Petersburger medicinische Wochenschrift .. .. .. .. | 1906, | 1909 |
| Surgery, Gynecology and Obstetrics .. .. .. .. | Vol. X | 1910 |
| Wiener klinische Wochenschrift .. .. .. .. .. .. .. .. .. .. | | 1909 |
| Zentralblatt für Chirurgie .. .. .. .. .. .. .. .. .. .. .. | | 1907 |
| Zentralblatt für Gynäkologie .. .. .. .. .. .. .. .. .. .. .. | | 1907 |
| Zentralblatt für innere Medicin .. .. .. .. .. .. .. .. .. .. | | 1907 |

### Local Medical Notes.

### UNIVERSITY OF BRISTOL.

**Congregation.**—The first congregation for conferring degrees of the University was held at the Victoria Rooms on October 20th, when the Vice-Chancellor was supported by the Deans of the Faculties, the Lord Mayor and Sheriff, and the Master of the Merchant Venturers.

**Convocation.**—At the first Convocation held on October 27th the Rev. de Lacy O'Leary, M.A., B.D., was elected Chairman, and Dr. Nixon as the representative on the Council.

**Court.**—At the meeting of the Court Dr. T. H. Warren was added to the members of the Council.

**New Physiological and Chemical Laboratories.**—The new Physiological and Chemical Laboratories at the University were officially declared open on November 15th. The ceremony began in the Great Hall with prayers, offered by the Bishop of Bristol, and the singing of two hymns. Addresses were then given by the Pro-Chancellor, Lord Winterstoke, and Dr. Herbert Warren, President of Magdalen College, Oxford (now a member of the University Council). A procession was then formed, headed by the Chairman of the Council, Mr. Lewis Fry, with the Lord Mayor, city officials, and members of the University in stately array. These passed round through the quadrangle by the road to the entrance of the new buildings, where a guard of honour of the University contingent of the Officers' Training Corps, under Major Swayne, was drawn up. Mr. Lewis Fry gave three knocks on the door with an ivory mallet, whereupon Lord Winterstoke from within the building unlocked the door, and announced the new wing open. On the same evening, in spite of bad weather, a most successful and well-attended reception was given in the new buildings. Many scientific demonstrations were prepared which were calculated to give the visitors some idea of the up-to-date equipment of the new laboratories. The University is to be congratulated on possessing two departments which represent the latest developments for teaching and research in physiology and chemistry. Space prevents our giving full details of all that the new laboratories contain. Their resources have been minutely described in the *British Medical Journal* of November 26th.

Professors Kent and Francis deserve the highest praise for the energy they have devoted to ascertain what equipment the best laboratories in England and abroad have found necessary, and for their sound judgment in installing the same. The buildings reflect the greatest credit also on the General Purposes Committee of the University, on the architect (Mr. Oatley), and on the builders (Messrs. Cowlin and Son), each and all of whom have contributed to adding to the University a new wing which encourages us to believe that the requirements of sound learning are understood and appreciated by everyone who has had a hand in the matter. Where money can be wisely expended it should be ungrudgingly bestowed. There still remain other departments in the University of Bristol to whose improvement men of wealth might contribute with every confidence that their money will not be wasted, but judiciously applied to the better advancement of knowledge.

**Association of Alumni.**—The first dinner of this Association was held on October 21st, under the presidency of Mr. Munro Smith. Sir Oliver Lodge was the guest of the evening, and in his speech pointed out the similarity in the origins of Birmingham and Bristol Universities.

**University Settlement.**—A meeting to promote the establishment of a University Settlement was held in the afternoon of October 21st, 1910. Sir Oliver Lodge was one of the chief speakers. The object of the institution is to promote the general welfare in its neighbourhood and to provide a centre for social study and training.

### EXAMINATION SUCCESSES.

Conjoint Board.—*Chemistry and Physics:* G. S. Terry. *Practical Pharmacy:* C. J. D. May, A. G. Fisher, C. N. Vaisey. *Medicine:* S. Marle, E. Christofferson. *Surgery:* C. Hart. *Midwifery:* G. H. Griffiths, R. F. Bolt, L. C. Watkins-Baker.

M.R.C.S., L.R.C.P.—Henry B. Parker.

L.D.S. Eng.—*Mechanical Dentistry:* P. L. Ealand.

### APPOINTMENTS.

**Bristol Royal Infirmary.**—H. Chitty, M.B., M.S. Lond., F.R.C.S., has been re-appointed Senior Resident Medical Officer and House Surgeon; R. C. Clarke, M.R.C.S., L.R.C.P., Casualty Officer.

**Bristol General Hospital.**—The following appointments have been made:—T. E. Malherbe, M.B., Ch.B. Edin., House Physician; A. W. Burton, M.B., Ch.B. Edin., House Surgeon;

P. J. Veale, M.B., B.S. Lond., Casualty House Surgeon; C. B. Tudehope, M.B., Ch.B. Edin., Assistant House Physician; Scott Williamson, L.R.C.P. & S. Edinb., Pathologist.

**Bristol Eye Hospital.**—To be Surgeons: C. H. Walker, F.R.C.S. Eng., M.B. Camb.; A. N. Godby Gibbs, M.R.C.S.; A. Ogilvy, M.D., F.R.C.S.I.

**Cossham Hospital.**—D. Robertson, M.B., Ch.B. Edinb., House Surgeon.

**Eye Dispensary.**—The following appointments have recently been made:—E. H. E. Stack, F.R.C.S., Surgeon, *vice* Dr. Ogilvy, resigned; A. Fells, M.B., C.M. Edin., W. P. Taylor, L.S.A., L.M.S.S.A., and H. B. Porteous, M.B., Ch.B. Edin., Assistant Surgeons.

**Brighton Royal Alexandra Hospital for Sick Children.**—W. Barrington Prowse, M.R.C.S., L.R.C.P., has been appointed Honorary Radiologist.

**Royal London Hospital for Diseases of the Chest.**—Latimer J. Short, M.D., B.S. Lond., Clinical Assistant.

**West Riding Asylum, Wakefield.**—Henry Devine, M.D., B.S. Lond., Deputy Superintendent.

---

**Philosophies.**—Dr. James Pearse, of Trowbridge, sends us the following note on "Philosophies," by Professor Ronald Ross:—

Few practitioners have time to indulge to any extent in the pleasures of literature; but all should seize an opportunity to peruse this small volume of verse by one of the leaders of the profession.

Here is a series of poems which reveal a sensitive mind stabbed by the poignancy of suffering multitudes, by the relentlessness of Nature in some of her moods, and earnestly seeking to read the riddle which such experiences provoke.

Briefly, the philosophy inculcates the pursuit of Science as the one certain means of lessening human travail, the necessity of thrusting aside all that does not pass the test of reason, of quitting dogma and speculation for solid fact.

> "Thee most we honour, thee,
> Great Science. Hold thy way:
> The end thou canst not see,
> But in the end the day.

We think it false to dream
Beyond the likely fact ;
We grant thee, Truth supreme,
Whatever thou exact."

One may not agree that such philosophy is the last word ; one may argue that man's nature comprises more than reason, that faith is more than fancy, that the conquest of Nescience will not alone rid the world of tribulation ; but one cannot but admire the whole-hearted persistence with which the author works out his creed—a creed which in his case has produced so beneficent an issue.

The work of the medical profession brings them day by day in contact with suffering ; the very repetition of it tends to dull the sense of sympathy. The wounds of the lame brother are bound, but the tragedy of his sufferings is not always appreciated. It is well to try to keep the lamp of sympathy burning while using all the powers of scientific application. Such combination of sympathy and practical effort the "Philosophies" pourtray with no light touch. The poverty-stricken multitudes of the east, decimated by famine and pestilence, stretch out appealing hands for help. It is this sense of tragedy which appears to have stirred Professor Ronald Ross to bend his mind unceasingly towards its alleviation. He shows us the tragedy of the Indian mother nursing a three-year child at her withered breast, of the corpse tossed on the Ganges flood, of malaria's "heated hands and fevered eyes."

"The painful faces ask, Can we not cure ?
We answer, No, not yet ; we seek the laws.
O God, reveal through all this thing obscure
The unseen, small, but million-murdering cause."

. . . . .

"Half stun'd, I look around
And see a land of death—
Dead bones that walk the ground,
And dead bones underneath.

A race of wretches caught
Between the palms of Need,
And rub'd to utter naught,
The chaff of human seed.

. . . . .

And this one, fever'd, flags,
And that one, hopeless, tries,
Or, uncomplaining drags
A giant leg, and dies."

The poems *In Exile* deal largely with experiences of desert life—the pitiless sun "a pestilence of light," the dust, the cry for rain, horrors of the night-time, the longing for the homeland. They contain many passages of striking power and vividness.

Such is the setting of Professor Ronald Ross's famous work—a life of eager, patient pursuit of Science in the midst of much personal travail, lived by a man consumed with the thought of the agonies of those amongst whom he moved, and the pitifulness of their lot. Of the consummation of his work he speaks thus :—

> " This day relenting God
> Hath placed within my hand
> A wondrous thing : and God
> Be praised. At His command
>
> Seeking His secret deeds,
> With tears and toiling breath,
> I find thy cunning seeds,
> O million-murdering death.
>
> I know this little thing
> A myriad men will save.
> O Death, where is thy sting ?
> Thy victory, O Grave ? "

It has been said that wherein modern life mainly surpasses that of the ancients is in the extension of sympathy and in the mass of attainable knowledge. It is to the credit of Professor Ronald Ross that he exemplifies the sympathy and has very materially added to the knowledge. He may be considered to have won the crown of Wordsworth's " happy warrior "—

> " Who, doomed to go in company with pain
> And grief and bloodshed, miserable train,
> Turns his necessity to glorious gain."

### INDEX.

Almond, Dr. G. H.—Notes on a case of oxycephaly, 118.
Amyotonia congenita, 45.
Bacillus coli, Infection of the urinary tract with—Dr. J. R. Charles, 23, 91.
Biliary and intestinal sand, Notes on—Dr. G. Parker, 112.
Blackwell, Dr. Elizabeth, Notes on the life of, 179.
Bladder, Treatment of growths of the, 49.

**Books, Reviews of—**
Achorn, Dr. J. W.—Nature's help to happiness, 168.
Age, incidence, sex, and comparative frequency in disease—Dr. J. G. Andrew, 158.
Air and health—Dr. R. C. Macfie, 168.
Allbutt and Dr. H. D. Rolleston, Sir T. C. [Eds.]—A system of medicine, 165.
American Dermatological Association, Transactions of the, 258.
Anæmias, Severest—Dr. W. Hunter, 247.
Anæsthesia, Golden rules of—Dr. R. Probyn-Williams, 69.
Andrew, Dr. J. G.—Age, incidence, sex, and comparative frequency in disease, 158.
Arthritis deformans—Dr. R. L. Jones, 153.
Aseptic surgery—C. B. Lockwood, 169.
Ash, Dr. E.—Mind and health, 168.
Aural surgery, A short practice of—Dr. J. A. Jones, 66.
Aural surgery, The operations of—C. E. West and S. R. Scott, 254.
Ballet, Dr. G.—Neurasthenia, 62.
Bardswell, Dr. N. D.—The expectation of life of the consumptive after sanatorium treatment, 167.
Barr, Drs. T. and J. S.—Manual of diseases of the ear, 69.
Bates, S. H.—Open-air at home, 71.
Bishop, E. S.—Lectures on surgical nursing, 259.
Blair, Dr. C.—Errors of refraction, 170.
Bosanquet and J. W. H. Eyre, Drs. W. C.—Serums, vaccines, and toxines in treatment and diagnosis, 359.
Bramwell, Dr. J. M.—Hypnotism and treatment by suggestion, 71.
Branson, Drs. H. Thursfield and W. P. S.—Medical morbid anatomy and pathology, 152.
Burghard, F. F.—A system of operative surgery, 160, 249.
Catalogue (Index-) of the library of the Surgeon-General's office, United States Army, 164.
Childhood, Common disorders and diseases of—Dr. G. F. Still, 248.
Childhood, Functional nervous disorders in—Dr. L. G. Guthrie, 61.
Clarke, Dr. H.—Studies in tuberculosis, 70.
Clarke, Dr. J. J.—Congenital dislocation of the hip, 357.
Clay, R. M.—The mediæval hospitals of England, 63.
Clinical medicine, System of—Dr. T. D. Savill, 67.

**Books. Reviews of** (*continued*)—
Clothing, Cerebral congestion and tight neck—Dr. W. G. Walford, 171.
Colon, Diseases of the—Dr. P. L. Mummery, 253.
Constipation and allied intestinal disorders—Dr. A. F. Hertz, 154.
Constipation, The operative treatment of chronic—Arbuthnot Lane, 68.
Consumption, its prevention and home treatment—Dr. H. H. Thomson, 167.
Consumptive after sanatorium treatment, Expectation of life of the—Dr. N. D. Bardswell, 167.
Corner, E. M.—Male diseases in general practice, 259.
Dawson, E. R.—The causation of sex, 166.
Deuteronomy Smith, 164.
Drainage, House, sewerage, and sewage disposal in relation to health—Dr. L. C. Parkes, 73.
Drug bill, How to cut the—Dr. A. H. Hart, 259.
Ear, Handbook of diseases of the—R. Lake, 170.
Ear, Manual of diseases of the—Drs. T. and J. S. Barr, 69.
Emergencies in general practice—Dr. P. Sargent, 169.
Emery, Dr. W. D'E.—Immunity and specific therapy, 252.
"Epilepsia," 360.
Epilepsy, Morison lectures on—Dr. W. A. Turner, 360.
Ethics, Medical—Dr. R. Saundby, 66.
Eye, The pathology of the—Dr. J. H. Parsons, 250.
Eyre, Drs. W. C. Bosanquet and J. W. H.—Serums, vaccines, and toxines in treatment and diagnosis, 359.
Gall, Dr. F. J., The unknown life and works of—Dr. Bernard Hollander, 69.
Gallenstein chirurgie, Drei jahre—Dr. H. Kehr, 162.
Glaister, Dr. J.—Poisoning by arseniuretted hydrogen or hydrogen arsenide, 58.
Gonorrhœa in the male, The treatment of—Dr. C. Leedham-Green, 56.
Gordon, Dr. W.—The influence of strong, prevalent, rain-bearing winds on the prevalence of phthisis, 167.
Gout—Dr. H. Strauss, 68.
Groves, E. W. Hey—A synopsis of surgery, 169.
Guthrie, Dr. L. G.—Functional nervous disorders in childhood, 61.
Habershon, Dr. S. H.—Diseases of the stomach, 64.
Hart, Dr. A. H.—How to cut the drug bill, 259.
Health, a royal road to it—J. P. Sandlands, 73.
Henry Phipps Institute for the study of tuberculosis, reports, 67, 171.
Herschell, Dr. G.—Soured milk and pure cultures of lactic acid bacilli in the treatment of disease, 73.
Hertz, Dr. A. F.—Constipation and allied intestinal disorders, 154.
Hip, Congenital dislocation of the—Dr. J. J. Clarke, 357.
Hollander, Dr. B.—The unknown life and works of Dr. F. J. Gall, 69.
Hospital nurse A—Memories, 362.

**Books, Reviews of** (*continued*)—
Hunter, Dr. W.—Severest anæmias, 247.
Hydrogen arsenide, Poisoning by—Dr. J. Glaister, 58.
Hypnotism and treatment by suggestion—Dr. J. M. Bramwell, 71.
Hysteria and allied vaso-motor conditions, Lectures on—Dr. T. D. Savill, 65.
Immunity and specific therapy—Dr. W. D'E. Emery, 252.
Infant, The nutrition of the—Dr. R. Vincent, 170.
Insanity in every-day practice—Dr. E. G. Younger, 361.
Jennings, Dr. O.—The morphia habit and its voluntary renunciation, 58.
Jones, Dr. J. A.—A short practice of aural surgery, 66.
Jones, Dr. R. L.—Arthritis deformans, 153.
Kehr, Dr. H.—Drei jahre gallenstein chirurgie, 162.
Lake, R.—Handbook of diseases of the ear, 170.
Lane, A.—The operative treatment of chronic constipation, 68.
Leedham-Green, Dr. C.—The treatment of gonorrhœa in the male, 56.
Leftwich, Dr. R. W.—Index of symptoms, 361.
Lejars, F.—Urgent surgery, 252, 358.
Lockwood, C. B.—Aseptic surgery, 169.
Logan, Dr. T.—Vetera et nova, 255.
Loose-leaf medical pocket-book, 360.
Lugaro, E.—Modern problems of psychiatry, 155.
Luke, Dr. T. D.—A manual of natural therapy, 62.
Macfie, Dr. R. C.—Air and health, 168.
Mackenzie, Dr. J.—Symptoms and their interpretation, 151.
Magennis, Dr. E.—Dictionary of ophthalmic terms, 67.
Male diseases in general practice—E. M. Corner, 259.
Marie, Dr. A. [Ed.]—Traité international de psychologie pathologique, 163.
Mediæval hospitals of England, The—R. M. Clay, 63.
Medical annual, The, 257.
Medicine, A system of—Sir C. Allbutt and Dr. H. D. Rolleston [Eds.], 165.
Mentally-deficient children—Drs. G. E. Shuttleworth and W. A. Potts, 359.
Mind and health—Dr. E. Ash, 168.
Morbid anatomy and pathology, Medical—Drs. H. Thursfield and W. P. S. Branson, 152.
Morphia habit and its voluntary renunciation—Dr. O. Jennings, 58.
Mummery, Dr. P. L.—Diseases of the colon, 253.
Muthu, C.—Pulmonary tuberculosis and sanatorium treatment, 157.
Natural therapy, A manual of—Dr. T. D. Luke, 62.
Nature's help to happiness—Dr. J. W. Achorn, 168.
Nervous disorders of children, Functional—Dr. L. G. Guthrie, 61.
Nervous system, Diseases of the—Dr. H. C. Thomson, 256.
Nervousness—Dr. A. T. Schofield, 168.
Neurasthenia—Dr. G. Ballet, 62.
Nursing, Surgical—E. S. Bishop, 259.
Open-air at home—S. H. Bates, 71.
Operationen, Rotter's typische, 72.
Operative surgery, A system of—F. F. Burghard, 160, 249.

**Books, Reviews of** (*continued*)—
Ophthalmic terms, Dictionary of—Dr. E. Magennis, 67.
Parkes, Dr. L. C.—House drainage, sewerage and sewage disposal in relation to health, 73.
Parsons, Dr. J. H.—The pathology of the eye, 250.
Pathological museum catalogue, University of Manchester—Dr. J. L. Smith, 258.
Photographic exposure record and diary, The "Wellcome," 165.
Phthisis, Influence of winds on the prevalence of—Dr. W. Gordon, 167.
Prescriber, The, 171.
Probyn-Williams, Dr. R.—Golden rules of anæsthesia, 69.
Psychiatry, Modern problems of—E. Lugaro, 155.
Psychologie pathologique, Traité international de—Dr. A. Marie [Ed.], 163.
Psychology and hypnotism, Experimental—Dr. G. H. Savage, 72.
Rat (The) and its relation to the public health, 362.
Rawling, Dr. L. B.—Landmarks and surface markings of the human body, 171.
Refraction, Errors of—Dr. C. Blair, 170.
Remedies, Some common—Dr. E. Smith, 73.
Renal function in urinary surgery, Estimation of the—Dr. J. W. T. Walker, 253.
Research defence society, Publications of the, 163.
Rhinology—Dr. P. W. Williams, 59.
Roller skating—J. Williams, 164.
Rolleston, Sir C. Allbutt and Dr. H. D. [Eds.]—A system of medicine, 165.
Ross, Dr. R.—Philosophies, 382.
Rotter's typische operationen, 72.
Sanatorium treatment of pulmonary tuberculosis—C. Muthu, 157; Dr. F. R. Walters, 57.
Sandlands, Dr. J. P.—Health, a royal road to it, 73.
Sargent Dr. P.—Emergencies in general practice, 169.
Saundby, Dr. R.—Medical ethics, 66.
Savage, Dr. G. H.—Experimental psychology and hypnotism, 72.
Savill, Dr. T. D.—A system of clinical medicine, 67; Lectures on hysteria, 65.
Schofield, Dr. A. T.—Nervousness, 168.
School children in Bristol, Report on medical inspection of, 256.
Scott, C. E. West and S. R.—Operations of aural surgery, 254.
Semmelweis: his life and doctrine—Sir W. J. Sinclair, 159.
Serums, vaccines, etc.—Drs. W. C. Bosanquet and J. W. H. Eyre, 359.
Sex, The causation of—E. R. Dawson, 166.
Shuttleworth and W. A. Potts, Drs. G. E.—Mentally-deficient children, 359.
Sinclair, Sir W. J.—Semmelweis: his life and doctrine, 159.
Sleeping sickness commission, Reports of 360.
Smith, Dr. E.—Some common remedies and their use in practice, 73.
Smith, Dr. J. L.—Pathological museum catalogue of Manchester University, 258.
Soured milk and pure cultures of lactic acid bacilli in the treatment of disease—Dr. G. Herschell, 73.
Sprains and allied injuries of joints—Dr. R. H. A. Whitelocke, 57.

**Books, Reviews of** (*continued*)—
Still, Dr. G. F.—Common disorders and diseases of childhood, 248.
Stomach, Diseases of the—Dr. S. H. Habershon, 64.
Strauss, Dr. H.—Gout, 68.
Surface markings—Dr. L. B. Rawling, 171.
Surgery, A synopsis of—E. W. H. Groves, 169.
Surgery, Urgent—F. Lejars, 252.
Symptoms and their interpretation—Dr. J. Mackenzie, 151.
Symptoms, Index of—Dr. R. W. Leftwich, 361.
Thomson, Dr. H. C.—Diseases of the nervous system, 256.
Thomson, Dr. H. H.—Consumption: its prevention and home treatment, 167.
Thursfield and W. P. S. Branson, Drs. H.—Medical morbid anatomy and pathology, 152.
Tuberculosis, Pulmonary, and sanatorium treatment—Dr. C. Muthu, 157; Dr. F. R. Walters, 57.
Tuberculosis, Studies in—Dr. H. Clarke, 70.
Turner, Dr. W. A.—The Morison lectures on epilepsy, 360.
Urgent surgery—F. Lejars, 252, 358.
Urinary surgery, Estimation of the renal function in—Dr. J. W. T. Walker, 253.
Vetera et nova—Dr. T. Logan, 255.
Vincent, Dr. R.—The nutrition of the infant, 170.
Walford, Dr. W. G.—Cerebral congestion and tight neck clothing, 171.
Walker, Dr. J. W. T.—Estimation of the renal function in urinary surgery, 253.
Walters, Dr. F. R.—The open-air or sanatorium treatment of pulmonary tuberculosis, 57.
West and S. R. Scott, C. E.—The operations of aural surgery, 254.
Whitelocke, Dr. R. H. A.—Sprains and allied injuries of joints, 57.
Williams, J.—Roller skating for health and pleasure, 164.
Williams, Dr. P. W.—Rhinology, 59.
Winds, Influence of, on prevalence of phthisis—Dr. W. Gordon, 167.
Younger, Dr. E. G.—Insanity in every-day practice, 361.

Boswell's *Life of Johnson*, The medical aspect of—Dr. B. M. H. Rogers, 289.
Bristol Medico-Chirurgical Society, 88, 185.
British Medical Association, Account of London annual meeting, 260.
Bromoform poisoning—Dr. Rupert Waterhouse, 129.
Brown, Dr. John, 265.

Cataract extraction, Operation for, 55.
Charles, Dr. J. R.—Infection of the urinary tract with bacillus coli, 23, 91; Report on medicine, 235.
Clarke, Dr. J. Michell—Cases of leukæmia treated by X-rays, 208.
Coe, R. W., Obituary notice of, 280.
Collins, C. H., Obituary notice of, 94.
Colour vision, Edridge-Green test for, 175.
Coombs, Dr. Carey—Diffuse destructive changes in the liver, associated with jaundice, 17; Report on medicine, 45.
Costobadie, H. P.—Hypnotism, 33.
Cotton, Dr. W.—Radiographic estimation of simple enlargement by means of visible shadows, 226.
Cutaneous eruption showing a bacillus morphologically corresponding with the Klebs-Lœffler, A case of anomalous—Dr. W. Kenneth Wills, 231.

Davidson, Dr. J. M.—Radium and some of its physical and therapeutic properties, 1.
Dermatology, Report on—Dr. J. A. Nixon, 246.

Editorial notes, 74, 172, 260, 363.
Epilepsy and its treatment, 140.

Fawcett, Dr. E.—The development of the human skull, 97.
Fibromyoma in pregnancy, 353.
Firth, Dr. J. L.—Report on surgery, 238.
Fletcher, Robert, 172, 190.
Fortescue-Brickdale, Dr. J. M.—Report on medicine, 140; Hyoscine and the mydriatic alkaloids, 317.
Fox (Long) Lecture, The, on the development of the human skull—Dr. E. Fawcett, 97.

Gangrene, Remarks on diabetic—C. A. Morton, 311.
Glaucoma, Treatment of chronic, 53.
Glycosuria, Remarks on the surgical aspect of—C. A. Morton, 311.
Goss, T. B., Obituary notice of, 379.
Green-Armytage, V. B.—Oriental medicine, stray jottings, 40.
Groves, E. W. H.—On the excision of strictures of the urethra, 325.

Hæmophilia, 347.
Hyoscine and the mydriatic alkaloids—Dr. J. M. Fortescue-Brickdale, 317.
Hypnotism—H. B. Costobadie, 33.

Hypophysectomy, 238.

Infantile paralysis, 343.
Insurance, State sickness, 366.
International post-graduate society, 174.
Intestinal sand, On biliary and—Dr. G. Parker, 112.
Iodoform and thyroidism—Dr. A. R. Short, 122.

Jaundice, Diffuse destructive changes in the liver associated with—Dr. C. Coombs, 17.
Jejunal ulceration, 91.
*Johnson*, The medical aspect of Boswell's *Life of*—Dr. B. M. H. Rogers, 289.

Klebs-Lœffler, A case of anomalous cutaneous eruption, showing a bacillus morphologically corresponding with the—Dr. Kenneth Wills, 231.
Knee-joint, Injury to the similunar cartilages of the, 144.
Koch, Dr. R., Notes on the life of, 177.

Labyrinthitis following mastoid operation, Acute, 147.
Laryngeal tuberculosis, Treatment by the galvano-cautery, 149.
Laryngology, Report on—Dr. P. W. Williams, 147.
Larynx, Cancer of the, of slow development, 148.
Leukæmia treated by X-rays, Cases of—Dr. J. M. Clarke, 208.
Library of Bristol Medico-Chirurgical Society, 85, 183, 274, 374; Annual report, 376.
Liver associated with jaundice, Diffuse destructive changes in the—Dr. C. Coombs, 17.
Local medical notes, 94, 188, 286, 380.

Medicine, Reports on—Dr. J. R. Charles, 235; Dr. Carey Coombs, 45; Dr. Fortescue-Brickdale, 140; Dr. G. Parker, 343.
Meningitis, Posterior basic, treatment of, 378.
Morgan, Presentation to Professor C. Lloyd, 76.
Morton, C. A.—A case of stone in the urethra, 127; Remarks on the surgical aspects of glycosuria, and on diabetic gangrene, 311.
Myasthenia with enlargement of the thymus, 377.
Myomata in pregnancy, 354.

Nixon, Dr. J. A.—Report on dermatology, 246.
Norman, Dr. H. J.—An old-time syphilidiater, 334.

Obituary notices, 93, 276, 379.
Obstetrics, Report on—Dr. W. C. Swayne, 352.
Ogilvy, Dr. A.—Report on ophthalmology, 53.
Ophthalmology, Report on—Dr. A. Ogilvy, 53.
Organotherapy in sclerodermia, 246. Oriental medicine, stray jottings—V. B. Green-Armytage, 40.
Oxycephaly, Notes on a case of—Dr. G. H. Almond, 118.

Parker, Dr. G.—Notes on biliary and intestinal sand, 112; Report on medicine, 343.
Pathological Society of Great Britain and Ireland, Bristol meeting, 286.
Polio-myelitis, Case of anterior, 90.
Poor law commission reports, and the medical profession, 78.
Post-graduate study—Dr. J. M. Rattray, 193.
Potassium iodide on tuberculosis of the skin, Congestive action of, 247.
Profily, John, 334.

Quinine as an abortifacient, 88.

Radiographic estimation of simple enlargement by means of visible shadows—Dr. W. Cotton, 226.
Radioscopy of the stomach, 346.
Radium and some of its physical and therapeutical properties—Dr. J. M. Davidson.
Rattray, Dr. J. M.—Post-graduate study, 193.
Remedies, Secret, 172.
Rogers, Dr. B. M. H.—The medical aspect of Boswell's *Life of Johnson*, with some account of the medical men mentioned in that book, 289.

Sclerodermia, Organotherapy in, 246.

Scrotum in the new-born, Sloughing of, 48.
Short, Dr. A. R.—Iodoform and thyroidism, 122; Report on surgery, 347.

**Sick, Preparations for the—**
Albulactin, 371.
Aperitol, 84.
Biogene diabetic biscuits, 371.
Bismuth gauze, Tabloid brand, 372.
Brussels Exhibition exhibits, 180.
Cascara-agar jelly, 273.
Ceregen, 370.
Digestin, 273.
Hordine, 371.
Hydropyrin, 181.
Iodine vasogen, 182.
Lactitia, 371.
"Macnair" automatic water steriliser, 271.
Meat port, 181.
Milk stout, 182.
Perhydrol, 372.
Potash-sulphur water, 273.
Roncegno, 182.
Salen, 84.
Sanitas-sypol, 272.
Sinapisms, 373.
Soloid bile salt, 270.
" bile salt agar-agar, 270.
" nutrient agar-agar, 270.
" " broth, 270.
" " gelatin, 270.
" " media, 269.
Sulphaqua, 272.
" medicated soap, 272.
Syrup cocillana compound, 373.
Tabloid Iodal, 271.
" pituitary gland, 83.
" thyroid gland, 83.
Thiocol, 372.
Thymoform tablets, 83.
Tuberculins, 268.
" and their uses, 271.
Vaccine coryza, 270.
" influenza, 270.
Vaporole ammonium chloride inhaler, 85.

Skull, The development of the human, Long Fox lecture on—Dr. E. Fawcett, 97.
Society, Bristol Medico-Chirurgical, 88, 185, 376.
Society of Great Britain and Ireland, Pathological, 286.
Spencer, W. H., Obituary notice of, 276.
Splenectomy, A brief account of—Dr. J. Swain, 223.
Stomach, Radioscopy of the, 346.
Surgery, Reports on—Dr. J. Lacy Firth, 238; Dr. J. Swain, 144; C. F. Walters, 49; Dr. A. R. Short, 347.
Swain, Dr. James—A brief account of splenectomy, with a case of simple hypertrophy of the spleen, treated by operation, 223; Report on surgery, 144.
Swayne, Dr. W. C.—Report on obstetrics, 352.
Syphilidiater, An old-time—Dr. H. J. Norman, 334.
Syphilis and aortic lesions, 47.

Thyroid and parathyroid glands, Functions of, 235.
Thyroid carcinoma, 237.
Thyroid inadequacy, 236.
Thyroidism, Iodoform and—Dr. A. R. Short, 122.
Tivy, W. J., Obituary notice of, 285.
Tobacco smoke, Nicotine and, 142.
Tuberculosis Conference at Edinburgh, 264.
Tumours of bilateral origin, Malignant and non-malignant, 92.

University of Bristol, 74, 363, 380.
Urethra, Case of stone in the—C. A. Morton, 127.
Urethra, On the excision of strictures of the—E. W. H. Groves, 325.
Urinary tract, Infection of the, with bacillus coli—Dr. J. R. Charles, 23, 91.
Uterine fibroids, 352.

Vitiligo, Experimental production of pigment in, 247.

Walters, C. F.—Report on surgery, 49.
Waterhouse, Dr. R.—Bromoform poisoning, 129.
Weatherly, F., Obituary notice of, 283.
Webster, T., Obituary notice of, 93.
Williams, Dr. P. W.—Report on diseases of the ear, nose and throat, 147.
Wills, Dr. W. K.—A case of anomalous cutaneous eruption, showing a bacillus morphologically corresponding with the Klebs-Lœffler and clearing up under diphtheritic antitoxin, 231.
Winsley sanatorium, 82, 191.
Wörger, R. G., Obituary notice of, 93.

X-ray treatment of laryngeal disease, 150.
X-rays, Cases of leukæmia treated by—Dr. Michell Clarke, 208.

